# COVID-19 pandemic and the global economic situation: a year on

**DOI:** 10.11604/pamj.2023.46.56.35920

**Published:** 2023-10-17

**Authors:** Kwame Adjei-Mantey, Anthony Amoah, Benjamin Amoah, Rexford Kwaku Asiama

**Affiliations:** 1 School of Sustainable Development, University of Environment and Sustainable Development, Somanya, Ghana

**Keywords:** COVID-19, health, macro-economy, recovery, global

## Abstract

A year after the World Health Organization´s declaration of the novel COVID-19 as a pandemic, the global macro-economic landscape has experienced severe shocks. As a result, developed and developing countries have been saddled with intense economic uncertainties. In this study, we discuss the global macro-economic environment during the pandemic declaration period and juxtapose it with the one-year post-pandemic declaration. The evidence shows significant negative impacts on macro-economic variables in the year of the declaration. However, signs of recovery are evident a year on, albeit slowly. To sustain and accelerate the recovery gains, we suggest that strategic macro-management policies are designed and strictly implemented. Anything short of this will see especially fragile countries plunged into an “economic abyss” with severe sociopolitical implications.

## Commentary

The COVID-19 disease, also known as the Severe Acute Respiratory Syndrome Coronavirus-2 (SARS-CoV-2), is alleged to have started in the city of Wuhan in China. It was not until March 2020 that the World Health Organisation (WHO) declared it a global pandemic. This declaration was occasioned by a 13-fold and a threefold increase in the number of cases and the number of countries with cases, respectively. The economic shock associated with the pandemic led to certain responses in the global macroeconomic environment in the short and medium-term [1,2]. Therefore, this study examines the macroeconomic impact a year after the COVID-19 pandemic declaration by the WHO. That is, how has the macroeconomic environment changed a year after the declaration of COVID-19 as a pandemic?

**Gross domestic product (GDP):** undoubtedly, COVID-19 has had an adverse effect on the global economy. Production suffered severely in many countries across the world, with supply-value chains disrupted globally. According to the World Bank, global gross output fell by an estimated 3.4% in 2020, with declining growth rates recorded in many regions across the globe. Sub-Saharan Africa´s regional economy (GDP) declined by 2.2% in 2020; Latin America and the Caribbean´s economy declined by 6.4%, Middle East and North Africa´s declined by 4.0%; and South Asia´s economy declined by 5.2% in 2020 [3]. In the East Asia and Pacific region, however, the economy grew by 1.2% in 2020 [3], which is a paltry rate of growth given the region´s high pre-pandemic growth rates. The economic decline recorded globally could be linked to border closures and domestic restrictions on mobility and production, thus disrupting supply value chains globally and, consequently, global output. Due to border closures in many parts of the world, industries suffered huge hits, with the aviation, hospitality and tourism industries being massively affected worldwide. Local restrictions, including lockdowns, also affected much of production in several countries. Even in countries where restrictions were not nationwide, they were imposed in local administrative areas with high population and high levels of business activity, thus severely hampering economic production. Additionally, the sheer number of people who got critically ill from COVID-19 infections also meant that critical labour was unavailable to work. While some industries were able to eventually shift part or all of their production online where workers who were not critically ill nor isolated could work remotely, it took some time for workers to adapt to that mode of work, thus losing crucial man hours. As a result, the GDP growth of many economies failed to meet anticipated pre-COVID levels. The economic situation was worse for developing countries because those countries have hardly automated work systems, and remote working was not the norm in these countries. Indeed, developing countries are set to suffer more from economic downturns resulting from COVID-19 due to inadequate resources and buffers. The United Nations Development Programme (UNDP) has, for instance, projected that at least $220 billion worth of income will be lost in developing countries as a result of COVID-19 . The consequence is an increase in the number of people who will be pushed into extreme poverty in these countries, with its associated inequalities. For example, since the outbreak of the pandemic, inter- and intra-country inequalities have worsened [4]. The foregoing provides evidence of the damning effect COVID-19 has had on production and the potential effects particularly for developing countries going into the future. A year later, signs of economic recovery are evident at the global level. Thus, the global economy picked up in the year 2021, with growth increasing to an estimated 5.5% [3]. This was largely due to the lifting of COVID-19 related restrictions and the restoration of global supply chains, driving production close to pre-pandemic levels. Sustaining this level of growth would be possible if there were no new waves of infections that might necessitate a re-introduction of COVID-19 related restrictions. To forestall an occurrence of new waves and the dire effects particularly for developing countries, easy access to vaccines is needed to ramp up vaccinations among larger sections of their populations. This way, new infections would be kept in check, and economic activities could continue without interruptions with associated wellbeing implications.

**Unemployment and labor:** labour and labour mobility in almost every country were affected by the COVID-19 pandemic. As explained in the previous section, due to reduced production, firms were forced to lay off workers during the period, especially if their operations made remote working impossible or non-feasible. As a result, unemployment increased in many countries, further worsening the plight of lower-income earners. The International Labour Organization (ILO) reports that 8.8% of global working hours were lost in 2020 [5]. This is the equivalent of 255 million full-time jobs, with regions such as Latin America and the Caribbean, Southern Asia and Southern Europe the hardest hit. Among those who stayed employed during the pandemic, working hours reduced, thus leading to lower incomes for them. Indeed, global income (not accounting for income support) declined by an estimated 8.3% in 2020, which is the equivalent of 4.4% of global GDP [5]. Given the fact that GDP growth suffered in many countries, economies shrank, and the prospects for new job openings going forward are even more limited. Furthermore, since the COVID-19 pandemic opened some firms to the possibility of remote working and the potential to achieve targets with limited physical infrastructure, such as office space, previous work requirements and conditions may likely not return for some of these firms. They may opt for operating with skeletal staff working remotely and may thus not need to employ peripheral staff such as office keepers, office security, front desk staff among others. This implies that high unemployment, particularly among low skilled personnel, is set to exacerbate. The International monetary fund [6] projected that losses in working hours would continue in the year 2021, with an average loss of 3.0% in global working hours which is the equivalent of 90 million full-time jobs. This was despite the economic recovery that was expected in the year 2021 and is testament to the fact that while production might return to pre-pandemic levels or close to that, it may not be accompanied by the full restoration of jobs that existed before the pandemic. In other words, employment recovery is set to lag behind output recovery, and this raises concerns for the future of work globally but more critically, in developing countries where relatively larger proportions of unskilled labor exist. A good starting point to restoring employment and work is to ensure mass vaccinations across countries. This will reduce the likelihood of infections among the labor force thus making them healthy with regards to COVID-19. This is a first step to making sure that labor is able to take up job openings should they become available. Furthermore, job creation should form a central part of the economic recovery policies across the globe. Such recovery policies ought to be robust and pay attention to production activities that can yield more jobs, especially for youth. Furthermore, low skilled workers who are trainable ought to be re-trained to be able to take up the jobs that may become available but require different set of skills than what they currently possess.

**Figure 1:**
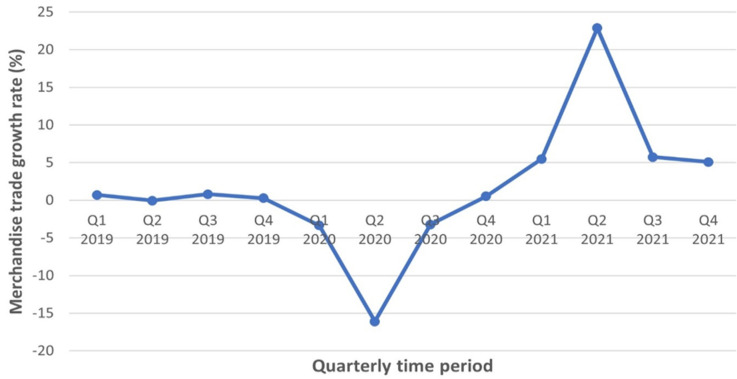
monthly world food [rice, sugar and maize] prices (Jan 2020-Mar 2022) source: United Nations Conference on trade and development

**Global trade:** following historical evidence of global shocks, the onset of the COVID-19 meant that there would be disruptions in global trade. Eventually, in 2020, commerce and output volumes fell to their lowest levels since World War II. In the first half of 2020, global industrial production and goods trade declined at a rate comparable to that seen during the depths of the Global financial crisis. However, they appeared and vanished more rapidly, indicating a stronger V-shaped recovery in 2020. The trends in global merchandise trade for 2020 and 2021 are presented in Figure 1. Generally, the effects of trade and production on specific items, services, and trading partners were highly variable. In fact, while the rest of the world suffered disruptions in trade, face masks were China's most exported product in May 2020. Exports in China fell sharply in February 2020, but soon rebounded and returned to normal by March 2020. However, shipments from the United States and Germany fell in April, recovering very slowly [7]. In addition, oil exports in African countries like Nigeria and Algeria fell by more than half while merchandise trade in many non-oil producing African countries changed marginally. Nonetheless, commerce in travel and tourism services also fell precipitously, while trade in digitally supplied services, such as telecommunications and information technology services, grew rapidly. It is estimated that in 2020, the value of goods exports reduced by 8.2% compared to the value of services exports, which declined by 16.7 percent [8]. By August of 2021, services and goods trade had recovered somewhat compared to the start of the epidemic, but new lockdowns and restrictive measures had caused more slumping. Recent trends in global trade point to some short to medium-term risk to global trade. This gives a bleak forecast for global trade in 2022. The invasion of Ukraine by Russia, new lockdowns in Shanghai and other parts of China over COVID-19 concerns make it difficult for suppliers to supply global demand for goods and services. Given that exports from Russia, China and Ukraine influence trade in other regions of the world, it is imperative that a solution is found to end the Russia-Ukraine war. Also, a cure for COVID-19 that is more robust might help end the uncertainties around jobs and help get economies working the way they were before the onset of the pandemic.

**Figure 2:**
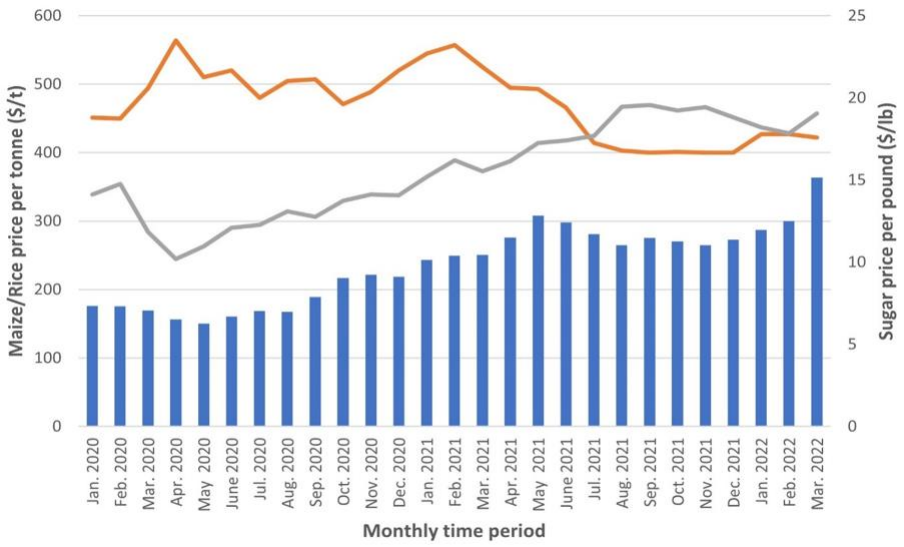
quarterly growth rate of global merchandise trade (Q1 2019-Q4 2021) source: United Nations Conference on trade and development

**Inflation:** in theory, one would expect the general price levels to rise in the face of sustained supply restrictions. These restrictions have adversely affected trade due to the rising cost of production which consequently forced general price levels to rise. Therefore, it is not surprising that there is inflation in food commodities globally. International food prices peaked in 2007-2008 and 2010-11. From Figure 2, it is obvious that food prices rose again in January-March 2022. The increase has sparked fears of a new global food crisis, resulting in increased hunger among the world's poor and, possibly, social upheaval. The increase in food prices is also observed in the Figure 2, which shows trends in the global market prices for commodities like rice, maize and sugar. Given the trajectory of the Food and Agriculture Organization (FAO) food price index and trend in global commodity prices from January 2022, it is reasonable to assume that prices for products will continue to rise. This is actually observed in the data on global prices of staples like rice, maize and sugar. A number of factors are probably driving inflation globally at this point in time. First, even though COVID-19 incidences have decreased significantly, the invasion of Ukraine by Russia has brought abruptions to global trade and caused supply shortages in countries that buy certain food commodities from either country. Both countries play an important role in the commerce of important foods like wheat, barley, corn, petroleum and other petroleum products. This makes it imperative that world policymakers find a solution to the impasse between these two countries and eliminate any associated supply chain disruptions. Secondly, according to harsh weather (hurricanes or drought) has produced shortages in oil supplies, coffee supply and microchip chips utilized in other smart technology. Because of Brexit and America´s tariffs on American imports, they argue, supplies to consumers in these countries and those elsewhere who rely on such commodities are being affected [9]. Finally, a number of countries have withdrawn the aid given to protect businesses and employment from the pandemic. It is possible that additional taxes will be implemented in order for governments to recoup their finances and promote fixed capital creation in the short- to medium-term, as a result. It is imperative that these triggers are addressed carefully in order to avoid a food crisis. The global economy will be better off without any subsequent crises.

**Financial markets:** the effects of the pandemic did not spare the world´s financial sector especially the fragile markets in developing countries. This has also, to a large extent, made the situation of poor households worse, as access to credit is anticipated to be tighter because of the pandemic and other related shocks. For example, the growth in domestic credit to the private sector as percentage of GDP for the world stood at 12.05 percent in the year 2020. In the absence of data, we used a five-year moving average to forecast estimates for 2021 and realise a 6.01 percent decline in domestic credit to the private sector. The reason is not farfetched. At the peak of the COVID -19 pandemic and the associated shutdowns of businesses, governments around the world had to support the private sector with special credit facilities to turn around businesses considering the critical nature of the private sector to growth and by extension economic wellbeing of the citizenry. As many economies around the world opened to businesses due to the easing of restrictions, the government credit support is expected to be reduced hence the decline. Similarly, on the capital market, the stock traded to GDP recorded a predicted growth of 24.61 percent, in the year 2020; this is expected to increase by 10.96 percent in the year 2021. The predicted growth could be attributed to GDP contractions in 2020, hence the stock value of stock traded assumed a large proportion of GDP. The predicted estimate for 2021 is plausible because most economies are on a rebound; hence, the value of stock traded would not weigh as high as that of the year 2020 relative to the predicted GDP growth. Using the market capitalisation to GDP measure, the ratio of the market capitalization of listed companies to GDP was 23.72 percent for the world in the year 2020, this is expected to decline sharply by 10.49 percent in the year 2021, possibly because many listed companies are recovering from the shocks of COVID-19 and are yet to record pre-pandemic year´s financial performance which would trigger investor buying sentiments. In addition, the rebound support to especially developing economies is expected to cause growth in world gross debt level by 13.59; this forecast is based on the International Monetary fund fiscal monitor [6].

## Conclusion

Admittedly, since the COVID-19 pandemic declaration, the global macroeconomic environment has not been spared its negative consequences. Although devastating, evidence from this study shows signs of recovery a year on. This study proposes strict targeted policies aimed at sustaining the recovery in addition to addressing the internal and external macroeconomic risks. If this is unattended to, many developing countries may experience one of the worst socio-eco-political crises ever.
